# Supplementary material for: The effect of intrapartum prolonged oxygen exposure on fetal metabolic status: secondary analysis from a randomized controlled trial
Source: Front Endocrinol (Lausanne). 2023 Jun 27;14:1204956. doi: 10.3389/fendo.2023.1204956 (PMC10335765; doi:10.3389/fendo.2023.1204956)
Supplement: Supplementary file 3 [file DataSheet_3.zip › Result-X101SC21092977-Z01-J001-B1-42/1.MetQuant-QC/Samples_neg-PCA.pdf]

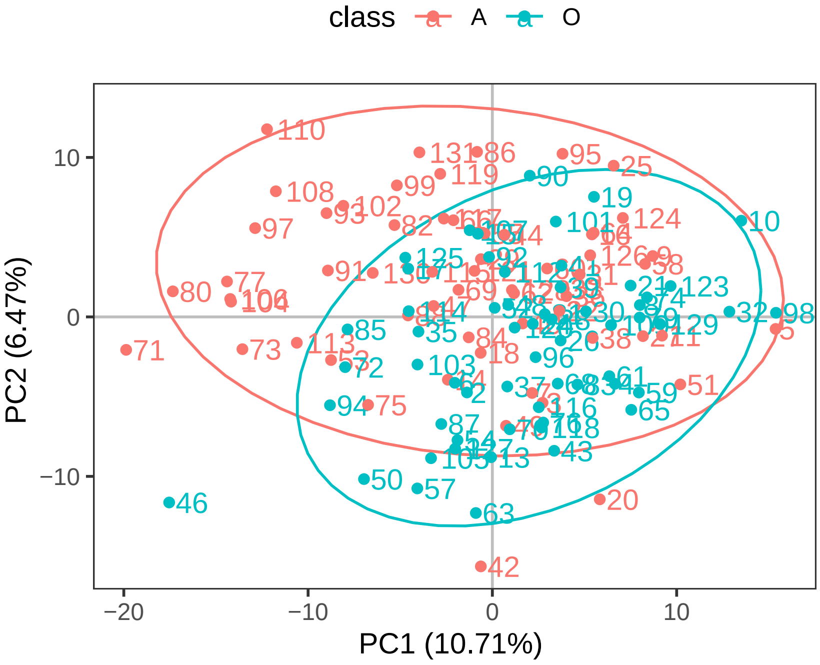

class
A
O
PC1 (10.71%)
PC2 (6.47%)
−20
−10
0
10
−10
0
10
110
131
86
95
25
90
119
99
108
102
19
93
97
82
101
124
10
125
91
130
80
77
104
71
73
113
85
35
103
72
94
75
84
18
96
37
116
65
59
51
61
87
43
118
13
105
50
57
46
63
20
42
123
32
98
129
21
126
38
